# Exploring the Association Between Hypothyroidism, Mean Platelet Volume, and Red Cell Distribution Width: A Case-Control Study

**DOI:** 10.7759/cureus.75927

**Published:** 2024-12-18

**Authors:** Mohammed Assaf Almalki, Abdullah M Alzahrani, Loay J Alghamdi, Mamdoh M Ahmed, Khalil M Alsulami, Mohammed M Alghamdi, Mustafa M Bukhari, Mulham A Kalantan

**Affiliations:** 1 College of Medicine, King Saud Bin Abdulaziz University for Health Sciences, Jeddah, SAU; 2 Department of Family Medicine, Ministry of the National Guard-Health Affairs, King Abdulaziz Medical City, Jeddah, SAU; 3 Research, King Abdullah International Medical Research Center, Jeddah, SAU

**Keywords:** case-control study, hypothyroidism, mean platelet volume (mpv), red blood cell distribution width (rdw), thyroid hormones

## Abstract

Background: Thyroid hormones are important in regulating hematopoiesis. Recent research has suggested that red cell distribution width (RDW) and mean platelet volume (MPV) may be useful inflammatory markers in various disorders, including thyroid disorders. Hence, the study aimed to evaluate the association between hypothyroidism, RDW, and MPV.

Methods: We used data from King Abdulaziz Medical City, Jeddah (KAMC-J) from May 2016 to June 2022. One hundred ninety-eight adults, 99 patients with hypothyroidism and 99 healthy controls, were assessed for thyroid function tests and a complete blood count (CBC). The association between hypothyroidism, RDW, and MPV was assessed using the Wilcoxon rank sum test. The sensitivity and specificity of RDW and MPV were plotted using the ROC curve.

Results: The median RDW levels of the hypothyroid and control groups were 14 (13-14.8)% and 13 (12.6-14)%, respectively (p<0.001). The median MPV levels of the hypothyroid and control groups were 10.43 (9.46-11.1) fL and 8.9 (8.47-10.1) fL, respectively (p<0.001). MPV levels greater than 9.40 have 77% sensitivity and 70% specificity in predicting hypothyroidism (AUC: 0.796 [95% CI: 0.735-0.858]; p<0.001). RDW levels greater than 13 have a sensitivity value of 75% and a specificity value of 51.5% in predicting hypothyroidism (AUC: 0.683 [95% CI: 0.608-0.758]; p<0.001).

Conclusion: Elevated mean platelet volume (MPV) and RDW levels were observed in individuals with hypothyroidism, suggesting that these parameters may serve as potential indicators of the condition. While this study identifies a significant association between MPV, RDW, and hypothyroidism, it does not conclude causality. Further investigation is warranted to determine the clinical utility of MPV and RDW as screening tools for hypothyroidism. These findings have potential implications for future research on the early detection and management of hypothyroidism.

## Introduction

Hypothyroidism, characterized by deficient production and release of thyroid hormones, particularly thyroxine (FT4), can lead to a range of symptoms, including cold sensitivity, slowed movements, constipation, and hair loss [1]. Hypothyroidism affects all body systems, as nearly all the body's cells have their metabolism, cell differentiation, and proliferation influenced by the thyroid hormones L-thyroxine (T4) and 3, 3', 5-L-triiodothyronine (T3) [2]. The thyroid-stimulating hormone (TSH) controls the production of these hormones through a negative feedback mechanism utilizing the hypothalamic-pituitary-thyroid axis [3].

Thyroid hormones are important for erythropoiesis through various mechanisms, including iron transport and utilization, as well as erythropoietin production and responsiveness [4]. They also play an essential role in megakaryocytopoiesis, which may be severely inhibited by myxedema in some cases of hypothyroidism [5].

The prevalence of hypothyroidism has been reported in several studies conducted worldwide. The prevalence of thyroid dysfunction in Saudi Arabia, particularly among women, is constantly increasing [6-8]. In a study conducted in the Hail region of Saudi Arabia, out of 175 patients, 98 cases (27 males and 71 females) were identified with hypothyroidism [9]. Furthermore, a study conducted in the Asir region of Saudi Arabia revealed a prevalence of 39.3% for subclinical hypothyroidism [10].

The red blood cell distribution width (RDW) and the mean platelet volume (MPV) are important components of the complete blood count (CBC), which are frequently underutilized [11]. RDW indicates anisocytosis and implies a homeostatic imbalance. MPV, on the other hand, implies an imbalance in megakaryocytopoiesis [11]. Because thyroid hormones play a role in both hematopoiesis and megakaryocytopoiesis, RDW and MPV may have diagnostic value.

The link between thyroid hormone and anemia is established in the literature [12-14]. A pooled analysis of 42,162 participants revealed an odds ratio of 1.84 to develop anemia in overt hypothyroid patients compared to euthyroid individuals [12]. Despite this, the relationship between RDW and thyroid status remains unclear. A study on 13,622 Chinese adults showed a non-significant association between RDW and thyroid status [15]. Another study on 6,895 American adults showed a significant association between RDW and TSH levels [16]. These contradictory results necessitate a further assessment of the relationship between RDW and TSH. The effect of thyroid hormones on platelet volume is controversial. A large study found that T4 levels but not T3 levels nor TSH levels are associated with platelet count, and there was no significant association between any of the thyroid hormones and MPV [17]. This study aimed to investigate the association between thyroid hormones, RDW, and MPV in a cohort of Saudi adults.

## Materials and methods

Study population

We used data from the information system of King Abdulaziz Medical City, Jeddah (KAMC-J) - BestCare 2.0 - to examine the relationship between thyroid hormones, RDW, and MPV. Between May 2016 and June 2022, KAMC-J diagnosed overt hypothyroidism in 522 subjects over the age of 18. The control group consisted of 1099 selected patients from KAMC-J who were considered normal based on the physical examination and laboratory test results. We excluded patients under 18 years old, those who did not have the results of thyroid function tests, and those who lost follow-up. The sample size was calculated by using a two-sided confidence level of 95%, a power of study of 80%, a ratio of unexposed to exposed in the sample of 1, a percent of unexposed with the outcome of 40, an odds ratio of 2.25, a percent of exposed with the outcome of 60, a risk/prevalence ratio of 1.50, a risk/prevalence difference of 20, and the formula of Kelsey [18]. The required minimum sample size was estimated to be 198, 99 patients in each group.

Study measurements

We measured blood counts, including RDW and MPV, using Alinity HQ serial version 5.0. The normal range for RDW was 11.5-14.5%. The normal range for MPV was 8-12 fL. We measured the TSH and thyroid panel using Architect Plus I2000. The normal range for TSH was 0.6-4.5 mIU/L, and the free T4 normal range was 9-19 pmol/L. Hypothyroidism was diagnosed when TSH was >4.5 mIU/L with normal or low-free T4.

Statistical analysis

Categorical data were presented as frequencies and percentages and compared with Pearson's chi-square test. The Shapiro-Wilk test was used to analyze the normality of the study's numerical variables. A Wilcoxon rank-sum test was used, and the variables were presented as a median and an interquartile range. The receiver operating characteristic (ROC) curve was used to illustrate the diagnostic abilities of MPV and RDW in detecting hypothyroidism. A p-value < 0.05 was considered statistically significant. The statistical analysis was performed using R software (version 4.2.2, RStudio, Boston, MA). This study specifically evaluated MPV and RDW as hematological markers in association with hypothyroidism.

Ethical consideration

Patients' data were illustrated using medical record numbers (MRNs). All the personal information of patients, other than the data collection sheet, was not collected. The collected data were only accessible to the researchers. The PI ensured that the subjects' privacy and confidentiality were guaranteed. No identifiers were collected, and all data, both hard and soft, was saved within the NGHA premises and accessible only to the research team. Approval was given by the National Committee of Bioethics, King Abdullah International Medical Research Center (KAIMRC) (IRB 1857/22).

## Results

A total of 198 individuals participated in the study, predominantly females (70.71%), with a median age of 45. Regarding the blood indices of the participants, the medians for white blood cell (WBC), platelet (PLT), red blood cell (RBC), hemoglobin (HGB), hematocrit (HCT), RDW, MPV, TSH, and free thyroxine (T4) were 6.75, 273, 4.70, 13.20, 40.10, 13.50, 9.62, 2.01, and 12.93, respectively (Table 1).

**Table 1:** Demographic and clinical characteristics of hypothyroidism patients compared to the control group *Median (IQR); n (%). ^#^Wilcoxon rank sum test; Pearson's chi-squared test. N: number; WBC: white blood cell; RBC: red blood cell; HGB: hemoglobin; HCT: hematocrit; MCV: mean corpuscular volume; MCH: mean corpuscular hemoglobin; RDW: red cell distribution width; PLT: platelets; MPV: mean platelet volume; TSH: thyroid-stimulating hormone; Free T4: free thyroxine.

Characteristic	N	Overall, N = 198^*^	Control group, N = 99^*^	Hypothyroidism, N = 99^*^	p-value^#^
Age	198	45 (34.25, 55.00)	41 (31.50, 51.00)	51 (38.00, 59.50)	<0.001
Gender	198				<0.001
Female		140 (70.71%)	48.00 (48.48%)	92 (92.93%)	
Male		58 (29.29%)	51.00 (51.52%)	7 (7.07%)	
WBC	198	6.75 (5.30, 8.00)	6.60 (5.40, 8.00)	7 (5.30, 8.00)	0.5
Neutrophil	198	3.28 (2.19, 4.24)	2.93 (2.15, 4.20)	3.58 (2.20, 4.34)	0.2
Lymphocyte	198	2.56 (1.94, 3.06)	2.56 (2.04, 2.94)	2.55 (1.91, 3.17)	0.9
Eosinophil	198	0.19 (0.10, 0.29)	0.21 (0.10, 0.32)	0.16 (0.11, 0.25)	0.2
Monocyte	198	0.49 (0.40, 0.62)	0.49 (0.41, 0.61)	0.49 (0.40, 0.62)	0.8
RBC	198	4.70 (4.30, 5.10)	4.90 (4.60, 5.30)	4.50 (4.19, 4.95)	<0.001
HGB	198	13.20 (12.10, 14.48)	14.00 (12.55, 15.15)	12.70 (11.70, 13.55)	<0.001
HCT	198	40.10 (37.50, 43.80)	43.10 (38.55, 45.70)	39.20 (36.00, 41.00)	<0.001
MCV	198	87.40 (82.73, 91.38)	88.00 (84.55, 91.10)	86.60 (80.65, 91.65)	0.2
MCH	198	28.35 (27.00, 29.90)	29.00 (27.55, 29.90)	28 (25.80, 29.95)	0.063
RDW	198	13.50 (12.70, 14.40)	13.00 (12.60, 13.95)	14 (13.05, 14.80)	<0.001
PLT	198	273 (233.00, 322.00)	266 (234.00, 311.50)	285 (228.00, 329.50)	0.4
MPV	198	9.62 (8.85, 10.70)	8.90 (8.48, 10.05)	10.43 (9.53, 11.10)	<0.001
TSH	198	2.01 (1.08, 3.51)	1.77 (1.17, 2.83)	2.74 (0.67, 5.51)	0.043
Free T4	198	12.93 (11.58, 14.59)	12.54 (11.47, 13.50)	14 (11.96, 16.01)	<0.001

The median age was significantly higher among patients with hypothyroidism compared to healthy individuals (p<0.001). Females had a higher risk of hypothyroidism compared to males (p<0.001). In patients with hypothyroidism, the median values for RBC, HGB, and HCT were significantly lower, while RDW, MPV, TSH, and free T4 were significantly higher compared to healthy controls (p<0.001) (Table 1).

**Figure 1:**
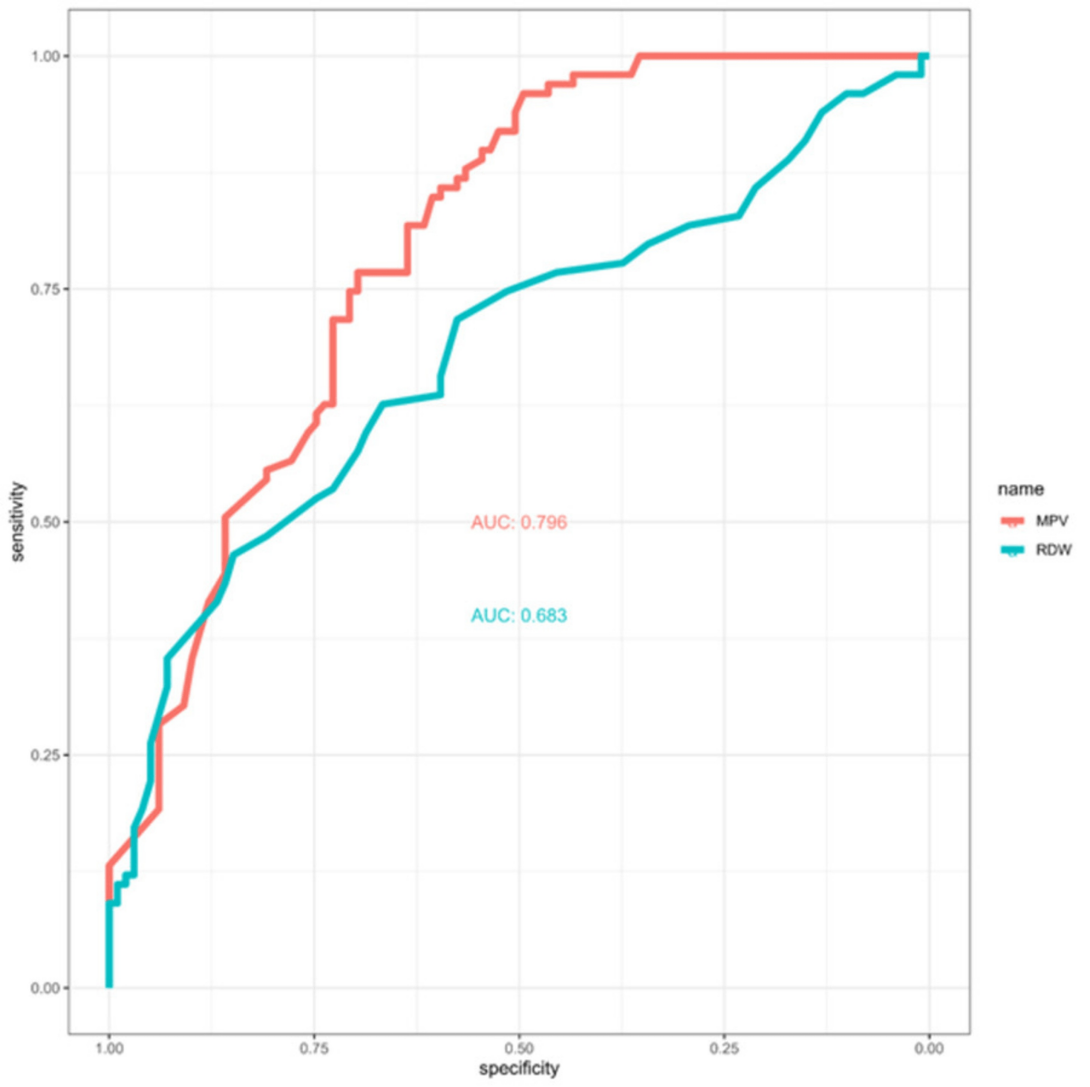
Diagnostic precision of MPV and RDW in predicting hypothyroidism using area under the curve MPV: mean platelet volume; RDW: red cell distribution width; AUC: area under the curve.

To optimize sensitivity and specificity, the cutoff values for MPV and RDW were determined as 9.67 and 13.6, respectively. The area under the curve (AUC) was 0.796 (95% CI: 0.735-0.858) for MPV and 0.683 (95% CI: 0.608-0.758) for RDW (Figure 1). The sensitivity was 0.717 and 0.626, and the specificity was 0.727 and 0.666 for MPV and RDW, respectively (Table 2).

**Table 2:** Mean platelets volume and red cell distribution width tests evaluation metrics *Using maximum sensitivity-specificity criteria. MPV: mean platelet volume; RDW: red cell distribution width; AUC: area under the curve.

Variables	MPV	RDW
Specificity	0.727	0.666
Sensitivity	0.717	0.626
AUC	0.796	0.683
95% CI	0.735–0.858	0.608–0.758
Positive predictive value	0.724	0.652
Negative predictive value	0.72	0.64
Cut off*	9.67	13.6

## Discussion

This study aimed to investigate the association between hypothyroidism and both RDW and MPV in Saudi individuals. Our findings demonstrated that individuals with hypothyroidism have higher levels of RDW and MPV compared to controls with euthyroid status. Both MPV and RDW were significantly associated with the presence of hypothyroidism in our study population.

In this study, patients with hypothyroidism were significantly older than euthyroid controls. The incidence of hypothyroidism is known to increase with age, which led some associations, like the American Association of Clinical Endocrinologists, to recommend screening of older patients [19,20]. Hypothyroidism was also prevalent among females, which is consistent with the literature and is partially explained by the autoimmune nature of thyroid dysfunction [21,22].

Regarding RBC indices, hypothyroidism patients were more likely to have a reduced RBC count, hematocrit, and hemoglobin level. It is established that hypothyroidism causes anemia [12-14], which may be due to bone marrow suppression, a reduced level of erythropoietin, comorbidities, or a concomitant deficiency of hematinics [14]. The MCV was not significantly different between the two groups. Normocytic anemia caused by erythropoietin deficiency is the most common form of anemia in hypothyroid patients [23]. However, hypothyroidism may also cause macrocytic anemia [24] and microcytic anemia [13], so the normal MCV may also be attributed to the multiple mechanisms involved in the pathogenesis and may be associated with anisocytosis [25].

RDW, which is calculated by dividing the standard deviation (SD) of the MCV by MCV, reflects anisocytosis [26]. It carries an important diagnostic value in differentiating types of anemia, especially the microcytic type [11]. It is worth noting that the RDW value changes before any change is noted in MCV [11]. In this study, the investigation revealed a significant increase in RDW among patients with hypothyroidism compared to controls with euthyroid status, which is consistent with the findings reported by Zhou et al. and Yu et al. [16,27]. The exact cause of this association is beyond the scope of our study, but other studies have suggested that anemia and inflammation play a role in this association [27]. Inflammation increases RDW, as noted in other studies investigating its association with autoimmune disorders [28,29]. This may explain the significant association between RDW and thyroid hormone in patients with Hashimoto thyroiditis [30]. In our study, RDW has a sensitivity of 62.6% for hypothyroidism, which is less than the 80% reported by Bilgin et al. The specificity, on the other hand, was 66.6%, which is higher than the 50% reported by them [1].

The inflammation does not only affect RDW but also MPV [31]. A pooled analysis of 6173 people found an increase in MPV in autoimmune thyroiditis [32]. It is also noted in several studies among patients with subclinical hypothyroidism [1,31-33]. Our study supported these findings, as MPV was significantly higher among patients with hypothyroidism. However, it contradicts other findings that suggest hypothyroidism is associated with a decrease in MPV [34] or no change in platelet size [35]. In a study done by Doormaal et al., the low sample size and the induced nature of hypothyroidism suggested that the results should be interpreted cautiously [34]. Though current evidence, including our study, suggests an increase in platelet size among patients with hypothyroidism, it is not clear if this is caused by a reduction of thyroid hormone or the inflammation present in autoimmune thyroiditis. This study was not powered to plot the exact association between thyroid hormone levels and MPV. The retrospective nature limited our ability to control for confounders like ESR and autoantibodies. Future studies should investigate the exact relationship between thyroid hormones and MPV. MPV was generally superior to RDW in detecting hypothyroidism, as indicated by higher sensitivity and specificity.

The findings of this study should be interpreted in light of some limitations. First, the study excluded patients with subclinical hypothyroidism, hyperthyroidism, and thyroid cancer, potentially limiting the generalizability of the results to these groups. Furthermore, this study did not assess iodine deficiency, which is a known factor in thyroid dysfunction and could influence the results. Lastly, while this study identifies an association between MPV, RDW, and hypothyroidism, it does not establish causality or predictive capacity, which requires further investigation in future studies with larger sample sizes.

## Conclusions

This study represents the inaugural investigation into the potential correlation between hypothyroidism, MPV, and RDW. Our findings revealed a notable association between hypothyroidism and altered levels of MPV and RDW. This observed variation may be linked to inflammatory processes observed in thyroiditis, which is the predominant etiology of primary hypothyroidism. Alternatively, we can attribute it to disturbances in hematopoiesis and megakaryocytopoiesis caused by diminished thyroid hormone levels. Drawing from our results, both MPV and RDW demonstrate promising utility as early indicators for the presence of hypothyroidism, with MPV exhibiting superior diagnostic potential. Consequently, it is recommended that future investigations explore alterations in these parameters following treatment and assess their prognostic significance.
